# Sensible Introduction of MR-Guided Radiotherapy: A Warm Plea for the RCT

**DOI:** 10.3389/fonc.2021.652889

**Published:** 2021-03-19

**Authors:** Helena M. Verkooijen, Lauren E. Henke

**Affiliations:** ^1^ Imaging and Oncology Division, University Medical Center Utrecht, Utrecht, Netherlands; ^2^ University of Utrecht, Utrecht, Netherlands; ^3^ Department of Radiation Oncology, Washington University School of Medicine in St. Louis, St Louis, MO, United States

**Keywords:** MR-guided radiotherapy, RCT, evaluation, evidence, MRgRT

## Abstract

Magnetic resonance guided radiotherapy (MRgRT) is the newest face of technology within a field long-characterized by continual technologic advance. MRgRT may offer improvement in the therapeutic index of radiation by offering novel planning types, like online adaptation, and improved image guidance, but there is a paucity of randomized data or ongoing randomized controlled trials (RCTs) to demonstrate clinical gains. Strong clinical evidence is needed to confirm the theoretical advantages of MRgRT and for the rapid dissemination of (and reimbursement for) appropriate use. Although some future evidence for MRgRT may come from large registries and non-randomized studies, RCTs should make up the core of this future data, and should be undertaken with thoughtful preconception, endpoints that incorporate patient-reported outcomes, and warm collaboration across existing MRgRT platforms. The advance and future success of MRgRT hinges on collaborative pursuit of the RCT.

## Introduction

Over the past several decades, the field of radiation oncology has witnessed a range of technical innovations. We have seen paradigm-shifting advances in planning techniques, like intensity-modulated radiation therapy (IMRT) and volumetric modulated arc therapy (VMAT). We have also seen the introduction of particle therapy with protons and carbon ions, with the potential to better-spare normal tissues, and improved precision of radiotherapy delivery with real-time tumor tracking (1). We have witnessed the introduction and adoption of advanced image-guided radiotherapy (IGRT) (2). Many, if not all of these innovations have (partly) replaced older techniques, some with good evidence for benefit (3, 4). However, in some cases, this has been without robust clinical evidence of superiority (5).

The newest of these technical advances to reach the mainstream clinic, magnetic resonance guided radiotherapy (MRgRT), offers real-time near-diagnostic visualization of the tumor/patient anatomy (6), enabling highly accurate online adaptive radiotherapy (ART), which by adjusting the treatment plan based on the daily anatomy while the patient remains on the treatment table, improves the dosimetric therapeutic index of radiation (7). This can be through both improved normal tissue sparing and/or accurate target dose escalation. Online ART with MRgRT has the potential to improve patient outcomes by reducing treatment-related toxicities and may enable increased local disease control through safe dose escalation. MRgRT also (presently) requires a more complex and resource-intensive workflow (8–10), with greater overall cost than standard RT.

The amount of clinical studies comparing MRgRT with CT guided radiotherapy is limited, and the number of completed or active randomized controlled trials (RCTs) even more so: in fact, we were unable to identify RCTs comparing CT guided radiotherapy with MRgRT, with the exception of one RCT in prostate cancer (clinicaltrials.gov NCT04384770.

Despite absence of strong evidence of improved efficacy, MRgRT is currently being implemented at many sites worldwide (11). In this paper, we argue that patients and societies can only truly benefit from this exciting new technology when the MRgRT community collaborates to generate solid clinical evidence, which, in large part, will require large, comparative randomized studies.

## Absence of Strong Clinical Evidence Is Bad for Patients

For many radiation oncologists, there is little doubt that MRgRT will improve patient outcomes. They argue that it is only logical that real-time target visualization, daily plan adaptation, and the option to accurately pause treatment during organ/tumor movement will lead to less irradiation of healthy tissue and therefore less toxicity, or better tumor control. Some would even say it is unethical to expose patients to less precise or accurate treatment in the context of a comparative study or a randomized controlled trial (RCT).

Yet, in medicine we have seen too many examples where the theoretical benefits of new treatments were not confirmed in clinical practice (12). There are also examples of promising new interventions which turned out to be harmful for patients (13, 14), or beneficial in particular settings (15) but harmful in others (16). This is even true in multiple examples where early Phase I and II clinical evidence of seemingly obvious and stepwise approaches suggested benefit (17), only to be proven wrong in RCT (18). Indeed, despite the typically promising early phase trial data that precedes an RCT, a shocking swath of Phase III oncology trials are negative according to primary endpoint (19). We should therefore pursue the highest possible level of evidence for MRgRT, and with collaborative enthusiasm.

The argument that an RCT is unethical in the setting of a “logically superior” technology also does not hold from the perspective of reimbursement and patient access to care. Although evidence is not the sole determinant of reimbursement decisions, mainstream reimbursement for a new technology is generally accelerated when high quality clinical evidence of superiority becomes available (20, 21). Reimbursement patterns in turn are related to access to particular types of care (22), as well as to outcomes of patients with cancer, even with efforts to control for comorbidities, stage, and similarly confounding variables (23, 24). Therefore, the earlier we enter or (even better) randomize some patients into control arms, the sooner many more patients may benefit when superiority is demonstrated.

Finally, from an economic perspective, it is also important to conduct high quality, randomized research. MRgRT is more expensive than most standard RT techniques, and like with any new promising technology, not all patients will benefit from MRgRT. There will be patients in whom the *a priori* risk of toxicity is so limited, that there is simply very little room for MRgRT to improve outcomes. Similarly, some patients will experience high toxicity despite MRgRT. Thus, from a cost perspective, we need to identify those patients and administer MRgRT only to patients who are likely to benefit.

## Why We Need Prospective Trials and Randomization, and Not Just Real-World Data

We need to demonstrate that theoretical benefits of MRgRT translate into real benefits for patients. As of today, the RCT remains the gold standard for demonstrating superiority of new treatments. Some argue that ‘real world evidence’, coming from large registries, can be a good alternative to RCTs. However, evaluation of new treatments using real world data is prone to a strong type of bias, i.e. confounding by indication. This type of bias is prevented by randomization.

Confounding by indication occurs in daily practice, where patients who are referred for a new, promising and innovative treatment like MRgRT are different from patients who are not. Usually, patients with access to innovative treatment are fitter, have less comorbidity, are more educated, have healthier lifestyles, and are better informed. Superior outcomes in these patients cannot be solely attributed to the new treatment, as they may very well be the result of difference in their pre-treatment health status and prognosis. These factors are generally difficult to measure and therefore impossible to (completely) adjust for by statistical analysis. With randomization, the treatment choice is based on chance only, and is independent of patient characteristics. Confounding by indication cannot be prevented in registry studies and real-world data, no matter how matter how big or how detailed they are.

## What Work Needs to Be Done Before We Can Embark on RCTs

Before a new technology like MRgRT is ready for formal comparison with the standard treatment, some preparatory steps need to be taken. A possible framework for this has been proposed is the R-IDEAL recommendations (25), based closely off of the IDEAL recommendations (26). The R-IDEAL concept describes the road towards evidence-based implementation of innovations in radiation oncology and starts with Stage 0 (Radiotherapy predicate studies), followed by stage 1 (Idea, first in man) and Stage 2a (technical development studies), after which clinical effectiveness of the technology is evaluated in early randomized controlled trials (Stage 2b, exploration, and Stage 3, assessment), followed by long-term study (Stage 4) and surveillance.

One important step is to develop a method for proper patient selection for trial entry. Not all patients are likely to benefit from MRgRT: some may have good outcomes with standard treatment, while in others the potential gain of MRgRT is so small that it will not translate into clinically meaningful or measurable improvements. An exceptionally elegant approach for patient selection that comes from the field of proton therapy, and may be readily applicable to MRgRT, is the model-based indication (27). Model-based indication is a stepwise methodology of selecting patients for a novel therapy when the primary goal is to reduce treatment-related toxicity. This approach comprises two phases, first to select patients who may benefit from a novel technology (MRgRT for this discussion) and second, to clinically validate MRgRT through comparative studies, preferably RCTs. In the first phase, patient selection is carried out by sequentially evaluating 1) normal tissue complication probability (NTCP) estimates for tissues of interest 2) *in silico* dosimetric comparison studies using MRgRT and ART vs. standard IGRT and conventional planning 3) the estimated clinical benefit based on the NTCP risk and potential dosimetric gains (28). Then, in phase two, patients with an expected clinical benefit (based on the phase one assessed NTCP-value reduction) that meets a defined clinical threshold will be enrolled in RCTs. Although this stepwise approach to clinical trial development may seem unusually structured (and perhaps aseptic), clinical development of MRgRT requires timely identification of best applications to minimize resource waste and to maximize the likelihood of long-term success.

Another area where pre-work is needed is the field of Health Technology Assessment (HTA). With health care costs rising disproportionally in many societies, payers and policy makers are, understandably, not always overly enthusiastic to adopt or reimburse new, more expensive interventions. Therefore, we advocate for groups to perform early health technology assessment, analyses where the costs per quality of life year gained (QALYs) are calculated and compared between technologies. In these models, assumptions of effectiveness and costs are made, in order to identify areas where MRgRT has the potential to become cost effective. These models will give insight, for example, into what extent toxicity needs to be reduced in order for MRgRT to become cost effective. Or, what the maximum costs of MRgRT are allowed to be, given a certain (likely) toxicity reduction. Conclusions of early HTA analyses may have varying implications across different countries worldwide, as the threshold for cost effectiveness varies internationally. Through early identification of scenarios where MRgRT will likely be too expensive or not incrementally effective enough to be cost effective, with mindfulness of international variation in cost-effectiveness, one can redirect research efforts to more worthy tumor sites or treatment strategies.

## What Endpoints Do We Need to Choose?

Choice of endpoint will depend on the stage of development of the technology. Of course, in early stages of technology and clinical development, more advantageous dose distributions, and lower dose to healthy tissues are encouraging and relevant. Smaller margins, adaptive planning, and more favorable dose distributions may indeed translate into lower toxicity of better quality-of-life. Similarly, dose-escalated treatment plans with sparing of organs-at-risk are likely to lead to better tumor control. However, we strongly believe that, at some point before widespread introduction of the technology, it is important to demonstrate that these theoretical benefits, confirmed by proxy endpoints, translate into real clinical benefits for patients. In the era of shared decision making, disease-free or progression-free survival are no longer the only or most important outcomes of interest. Neither are doctor-assessed acute and chronic toxicities.

We think that patients could, and should, play an important role in relevant trial endpoints. Also, we believe that patients themselves are in the best position to provide these endpoints. There is no one better positioned to report outcomes in the domains of physical functioning, role and social functioning, and cognitive functioning than a patient themselves. Patient reported outcomes (PROs) must be considered in MRgRT. Also, in terms of “traditional” outcomes, like measured toxicity, instead of the doctor taking a snap-shot at the outpatient clinic, we believe that toxicity and functioning are better assessed by the patients themselves at multiple time points during follow-up. Fortunately, multiple technological solutions (including established cloud-based solutions, apps, and websites) and tools (such as the validated PRO-CTCAE, EORTC QLQ-C30, and other questionnaires) are readily available (29–32). As MRgRT study designs are considered, strong consideration should be given to PRO-based endpoints as either primary or complementary objectives.

## Do We Always Need to Do an RCT?

As clear as our plea for the RCT in MRgRT may be, there are clinical scenarios in which RCTs are impractical and unnecessary. One such scenario is the evaluation of late effects. It remains a challenge to establish high quality late toxicity data, particularly when toxicities occur outside of standard clinical trial windows. In this setting, large, high quality, multi-institutional registries could play a pragmatic role in capturing a diversity of potential events that could occur sporadically and take place years following treatment completion. Given the potential rarity, diversity, and variably long timeframe for late toxicities to develop, they are an impractical endpoint for the clinical development of a novel technology. We need evidence for MRgRT imminently, and choices of late toxicities as primary endpoints for initial RCTs in the field would unreasonably delay clinical development. Similarly, RCTs for rare tumor sites where accrual is challenging and slow, remain impractical in the initial development of MRgRT. Registries may again be useful in this scenario. We would also argue that within particular body-site settings, gains through technology advances like MRgRT are often translatable across histologies. It stands to reason that if MRgRT were proven beneficial in toxicity reduction of SBRT for unresectable pancreatic cancer, it might similarly offer toxicity reduction for SBRT to a renal cell carcinoma oligometastasis to the pancreas.

Apart from late outcomes and rare cancers, it has also been argued many times that RCTs are inappropriate for “parachute” style situations. One would not perform an RCT of a parachute use vs. jumping without.

Of course, there may be individual situations where the anticipated benefit of MRGRT is too large to justify randomization. However, in general, like most medical practices, MRgRT is unlikely to present “parachute” style scenarios (33). One does not need to look far into the history of medicine to identify RCTs where the primary endpoints were thought by many to be slam dunks, but were indeed ultimately negative (34). Or, for that matter, where unexpected effects of an experimental approach decimated any benefit (35). We maintain that RCTs are the gold standard in oncology trials and non-randomized and observational studies should not be viewed as a replacement for them, but rather as a complement to them in the pursuit of MRgRT development.

## Can Varying MRgRT Treatment Platforms Be Used for a Common Goal?

In the clinical mainstream of MRgRT, two MR-linacs (MRL) platforms are commercially available and in global use. They are the 1.5-Tesla (T) MRL (Elekta Unity) and the 0.35T MRL (Viewray MRIdian). Although the imaging units on board these two MRLs vary in strength, the clinical imaging utility itself is similar with regards to clinician ability to distinguish the daily anatomy, even in complex soft tissue sites like the abdomen (36). Indeed, we believe these systems are far more alike than different, especially when placed in the context of other existing linacs. We do recognize there may be particular niche applications focused on imaging endpoints that may be best performed on one platform vs the other for consistency (37). However, the broad capabilities of the MRL systems, like online adaptation (whether it is referred to as “SMART” or “adapt to shape”) or MR-guided alignment and gating (MR-based setup, whether given the name “adapt to position” or not) are mainly translatable across platforms, with no greater difference than that between an Elekta Versa and a Varian Truebeam (the modern, high-throughput CT-based linacs at time of this writing). There is indeed long-standing precedent in the field of radiation oncology to permit multiple disparate technologies within a single trial, even with different imaging types, different multi-leaf collimators, and different motion management, as long as the delivered therapy is overall equivalent. To say that the 1.5T and 0.35T systems cannot be used interchangeably in a trial of online adaptation would be like saying a lung SBRT trial could only occur on a Truebeam, but not a tomotherapy or Cyberknife unit (38). Focusing on differences between MRLs, rather than similarities, will only divide efforts, attention, funding, and patient resources, and ultimately delay the success of MRgRT. Thus, RCT and other study efforts should aim to be collaborative across platforms and institutions, to maximize the timely impact of this new technology. Both platforms have formed consortia, where radiation oncologists, physicists, methodologists and other experts collaborate to work towards evidence-based implementation of the technology and optimized radiation treatment approaches to improve patients’ outcomes (39). These consortia are in the excellent position to design and initiate international, platform agnostic, multicenter RCTs.

Finally, it will be challenging to find the right balance between having enough sites offering MRgRT to run RCTs, while avoiding large scale uptake of the technology withouth clinical evidence. As with many technical innovations, the time window for RCTs is narrow. We should avoid a situation where MRgRT has been implemented on a large scale, where radiation oncologists and therapists have become accustomed to providing MRgRT, and where it has become too late to de-implement the technology for tumor sites of patient categories where RCTs have not been able to confirm superiority or cost-effectiveness. Therefor, it is imperative to start RCTs sooner rather than later.

## Conclusion

We believe that RCTs are central to the future success of MRgRT. Randomized data will help to identify and substantiate the potential clinical gains of MR-guidance, and will ensure coordinated dissemination of this novel technology. The MRgRT community needs to unite across platforms to enable thoughtful conception of randomized trials, with modern endpoints, and with timely generation of the high-quality evidence needed to support the future of the field.

## Data Availability Statements

The original contributions presented in the study are included in the article/supplementary material. Further inquiries can be directed to the corresponding author.

## Author Contributions

HV: Conceptualization and writing LH: Conceptualization and writing. All authors contributed to the article and approved the submitted version

## Conflict of Interest

HV reports research support from Elekta. LH reports (research) support from Viewray.
